# Clinical effects of central antagonist of cholecystokinin-1 receptors GB-115 in patients with Generalized Anxiety Disorder

**DOI:** 10.1192/j.eurpsy.2022.981

**Published:** 2022-09-01

**Authors:** O. Dorofeeva, T. Syunyakov, M. Metlina, N. Ivashkina

**Affiliations:** FSBI “Zakusov Institute Of Pharmacology”, Department Of Pharmacological Genetics, Moscow, Russian Federation

**Keywords:** anxiety disorder, cholecystokinin, anxiolytic

## Abstract

**Introduction:**

The pilot clinical study of GB-115, a new peptide antagonist of central cholecystokinin-1 receptors, revealed that drug was clinically effective in patients with generalized anxiety disorder (GAD) at dose 6 mg daily. Here, we provide results of post-hoc analysis of changes of anxiety and fatigue symptoms to give characterization of its clinical effects in clinically relevant doses.

**Objectives:**

To research the changes of anxiety- and fatigue-related symptoms during GB-115 treatment in patients with generalized anxiety disorder (GAD).

**Methods:**

Patients with GAD without somatic diseases aged 18-55 years were eligible in the study. Patients were prescribed with GB-115 6 mg daily for 21 days. Anxiety and fatigue symptoms were assessed with Hamilton Anxiety Rating Scale (HARS) and Multidimensional Fatigue Inventory (MFI-20). Variables are described as medians and interquartile range (IQR). Pre-post comparisons were performed using the Friedman ANOVA at 2-side p-value <0.05.

**Results:**

25 patients diagnosed with GAD (8 males, 17 females; median [IQR] age: 34 [29.75, 43.0]) included in the analysis. Median [IQR] HARS total score decreased from 22 [20, 24.5] to 19 [16, 20], 13 [10.5, 15.5], 9 [5.5, 11] and 5 [3.5, 8] on the Day 3,7,14 and 21, respectively (χ2=95.07, df=4, p<0.001). Median [IQR] MFI-20 score decreased from 70 [46, 75.5] to 59 [41, 74.5], 52 [37.5, 64.5], 37 [26.5, 63] and 28 [24, 48.5] on the Day 3, 7, 14 and 21, respectively (χ2= 55.41, df=4, p<0.001). None of patients had stimulation-related side effects.

**Conclusions:**

GB-115 action in the treatment of GAD patients is characterized with anxiolytic action with mild psychostimulant properties.

**Disclosure:**

No significant relationships.

